# Conceptual Framework for Insomnia: A Cognitive Model in Practice

**DOI:** 10.3389/fnins.2021.628836

**Published:** 2021-07-22

**Authors:** Zahra Vaziri, Mohammad Nami, João Pereira Leite, Alexandre Cláudio Botazzo Delbem, Miguel Angelo Hyppolito, Iman Ghodratitoostani

**Affiliations:** ^1^Neurocognitive Engineering Laboratory (NEL), Institute of Mathematics and Computer Science, University of São Paulo, São Carlos, Brazil; ^2^Department of Neuroscience and Behavioural Sciences, Ribeirão Preto Medical School, University of São Paulo, Ribeirão Preto, Brazil; ^3^Neuroscience Center, Instituto de Investigaciones Científicas Servicios de Alta Tecnología (INDICASAT AIP), Panama City, Panama; ^4^Department of Neuroscience, School of Advanced Medical Sciences and Technologies, Shiraz University of Medical Sciences, Shiraz, Iran; ^5^Dana Brain Health Institute, Iranian Neuroscience Society-Fars Chapter, Shiraz, Iran; ^6^Academy of Health, Senses Cultural Foundation, Sacramento, CA, United States; ^7^Department of Cognitive Neuroscience, Institute for Cognitive Science Studies (ICSS), Pardis, Iran; ^8^Reconfigurable Computing Laboratory, Institute of Mathematics and Computer Science, University of São Paulo, São Carlos, Brazil; ^9^Department of Ophthalmology, Otorhinolaryngology, Head and Neck Surgery, Ribeirão Preto Medical School, University of São Paulo, Ribeirão Preto, Brazil

**Keywords:** cognitive model, insomnia, evaluative conditional learning, mediator model, distorted perception, appraisal, valence, conceptual cognitive framework

## Abstract

Insomnia is a widespread neuropsychological sleep-related disorder known to result in various predicaments including cognitive impairments, emotional distress, negative thoughts, and perceived sleep insufficiency besides affecting the incidence and aggravation of other medical disorders. Despite the available insomnia-related theoretical cognitive models, clinical studies, and related guidelines, an evidence-based conceptual framework for a personalized approach to insomnia seems to be lacking. This study proposes a conceptual cognitive framework (CCF) providing insight into cognitive mechanisms involved in the predisposition, precipitation, and perpetuation of insomnia and consequent cognitive deficits. The current CCF for insomnia relies on evaluative conditional learning and appraisal which generates negative valence (emotional value) and arousal (cognitive value). Even with the limitations of this study, the suggested methodology is well-defined, reproducible, and accessible can help foster future high-quality clinical databases. During clinical insomnia but not the neutral one, negative mood (trait-anxiety) causes cognitive impairments only if mediating with a distorted perception of insomnia (***Ind-1*** = 0.161, 95% CI 0.040–0.311). Further real-life testing of the CCF is intended to formulate a meticulous, decision-supporting platform for clinical interventions. Furthermore, the suggested methodology is expected to offer a reliable platform for CCF-development in other cognitive impairments and support the causal clinical data models. It may also improve our knowledge of psychological disturbances and complex comorbidities to help design rehabilitation interventions and comprehensive frameworks in line with the “preventive medicine” policies.

## Introduction

Behavioral sleep disturbances are classified into various types of insomnia, excessive daytime somnolence (EDS), sleep phase disorders, and parasomnias. These are potentially rooted in psychophysiological, cognitive, emotional, and behavioral abnormalities resulting in impaired sleep efficacy, disintegrated sleep cycles, and/or arousal instability (Cormier, 1990; Sateia, 2014). Insomnias are characterized by poor subjective sleep quality, difficulty in falling asleep and maintaining sleep at bed-time, wakes after sleep onset (WASO), or unprompted early morning awakening. The consequent diurnal symptoms may then present as inadequate cognitive functions, declined cognitive aptitude, fatigue, hampered productivity, depression or irritability, impaired decision-making, low motivation, and mood dysregulation (Mai and Buysse, 2008; Nami, 2014).

Recently, cognitive-vulnerability models, which theoretically justify the interrelation between sleeplessness and mood dysregulation or cognitive insufficiencies, have drawn the attention of the research community. When insomnia becomes a chief complaint, the vicious cycle of insomnia-anxiety-insomnia starts to emerge. Undeniably, affective dysregulation, impulsivity, restlessness, EDS, disrupted vigilance, and cognitive decline are some consequences of long-term sleep insufficiency in many instances (Nami, 2014).

Among the theoretical and cognitive-computational models related to insomnia, the cognitive vulnerability model for insomnia induced mood disturbances (CVMIMD), the sleep-specific cognitive vulnerability (SSCV), the behaviorally induced insufficient sleep syndrome with restricted and extended sleep opportunity (BIISS-RESO), and the global cognitive vulnerability to insomnia (GCVI) (Bei et al., 2015) are the main highlights. These models are addressed subsequently.

### Cognitive Vulnerability Model for Insomnia Induced Mood Disturbances

From the neurocognitive standpoint, the prefrontal cortex (PFC), which plays a pivotal role in affect-regulation and cognitive-control, develops intensely throughout the neurodevelopment phase and adolescence owing to neuroplasticity. When the hypnic tone is decreased either due to poor sleep hygiene or socio-behavioral and psychophysiological stressors, a proposed explanation is the activation of PFC’s maladaptive processes as a potential neurocognitive mechanism underlying the affective consequences of insomnia and inefficient sleep, in general (Freeman et al., 2005).

The body of psycho-behavioral and neurocognitive empirical evidence describing the precise mechanisms that underlie the link between insomnia and negative mood is thin. However, subjective sleep insufficiencies and dysregulated mood observations exhibited more robust relationship as compared to objective findings from polysomnography or even full-setup sleep electroencephalography data. This points to the fact that psychological factors that hinder sleep efficiency might play significant roles in justifying the sleep-mood crosstalk. Yet some of these insomnia-related cognitive vulnerability factors are now acknowledged as erroneous beliefs, cognitive biases, and thought patterns that increase the likelihood of the predisposed individuals toward psychopathology (Freeman et al., 2005).

### Sleep Specific Cognitive Vulnerability

In some instances, the erroneous beliefs and attitudes represent exclusive sleep-related problems in which case, the distressing worries related to insomnia-continuation are usually evaluated using the dysfunctional beliefs and attitudes about sleep (DBAS) Scale. Harvey’s cognitive model (Harvey, 2002) described the impact of the DBAS-related cognitive vulnerability on insomnia complaints. According to this model, insomniacs are generally worried about poor sleep and its daytime consequences, and such strong, negatively toned thoughts trigger selective attentional-emotional bias, wherein individuals over-monitor their sleep-related threat cues. Previous investigations proposed a strong connection between DBAS and poor sleepers, which happens to play a key role in DBAS-driven disturbances in sleep perception and sleep safety behaviors such as napping (Harvey, 2002).

### Behaviorally Induced Insufficient Sleep Syndrome With Restricted and Extended Sleep Opportunity

This condition refers to a typical complaint reported by the patients as “*at nights I cannot sleep, in the morning, I cannot wake up*.” Habitual sleep episodes are usually shorter (confirmed by history, sleep log, or actigraphy) for patients experiencing initial or maintenance insomnia compared to the normative values from age-adjusted groups. Such patients also report sleep-inertia in the morning and complain about EDS for a minimum of 3 months before the interview. However, they tend to sleep considerably longer on weekends or during vacation. In general, the reported objective sleep efficiency as detected by polysomnography is below 80%, besides the mean initial nocturnal sleep latency which takes a longer time, more than 45 min. Also, these patients report repeated WASOs (Bastien et al., 2008).

### Global Cognitive Vulnerability to Insomnia

Cognitive vulnerability is defined as global when the dysfunctional beliefs and attitudes are general and not necessarily focused on a distinct behavioral or experiential area. According to Beck’s cognitive model, psycho-traumas in the early years of life combined with a complicated past can foster negative attitudes and biases concerning both self, world and the future. Such beliefs yield maladaptive schemas that may trigger cognitive vulnerabilities and negative tendencies based on depression later in life (Beck, 2008). A few studies on GCVI suggest strong links between sleep predicaments (mainly insomnias) and negatively toned cognitive constructs. For instance, complaints of chronic insomnia in young adults were found to be associated with anxiety and depression-related cognitive factors (Alfano et al., 2009). In the same vein, Sadler et al. (2013) claimed hopelessness, a global cognitive-vulnerability factor in older adults, can amplify the effects of insomnia on depressive symptoms (Sadler et al., 2013).

The various types of insomnia (more than 10) require personalized treatment approaches. Some of the broadly described types include adjustment insomnia, drug or substance-induced insomnia, comorbid insomnia, onset insomnia, middle insomnia, late insomnia, conditioned, or psychophysiological insomnia, behavioral insomnia of childhood, idiopathic insomnia, paradoxical insomnia, and sleep hygiene insomnia (Dzierzewski et al., 2018). Based on the severity of sleep insufficiency, we have categorized insomnia patients into Neutral (with a mild to moderate perception of sleep difficulties) and Clinical (with a severe perception of sleep difficulty) types.

Gross (1998) designed the *modal model of emotion* as a conceptual framework to illustrate how emotions can be generated and evolve over time. The emotion-generation process begins with internal or external goal-relevant situations that draw attention to specific features of the situation, appraisals emerge to make meaning of the situation resulting in multi-faceted emotional responses and feedback to modulate the current situation perpetually. Collectively, the modal model reflects the dynamic nature of the emotions and suggests possible emotion-regulation strategies comprising of situation selection, situation modification, attention deployment, cognitive change, and response modulation (Gross, 2013).

To the best of our knowledge, the aforementioned studies have little mention of causality (mediation) relationships, which can easily mislead interpretations of the findings. Thus, necessitating the design of an approach to conceptualize these theories and hypotheses. A novel insomnia theoretical-conceptual framework would enable the drawing of data models for testing mediational relationships between independent variables and outcomes within retrospective studies. Besides, it may also help suggest research strategies and predictions designing prospective studies on insomnia. The present study aims to fill this void in the literature by proposing and validating a novel conceptual cognitive framework (CCF) for insomnia in light of the above-mentioned models. The CCF illustrates how cognitive processes and their interactions can generate annoyance-distress reactions, which in turn, lead to the development or maintenance of insomnia. The insomnia numerical model is also demonstrated through multi-mediatory (causality) modeling approaches.

## Proposed Conceptual Cognitive Framework

### Fundamental Ideas and Postulations of the Conceptual Cognitive Framework

• Conceptual cognitive framework aims at illustrating the interaction between cognitive processes that cause annoyance-distress reactions in insomnia.
• Conceptual cognitive framework rests mainly on evaluative conditioning, assuming a conscious attended awareness perception (CAAP) to both unconditioned stimulus (US) and conditioned stimulus (CS), and their contingencies essential for attitude formation.
• Either or both, cognitive-value and emotional-value, can cause annoyance; however, they can also affect each other merely through annoyance. Furthermore, annoyance distorts the corresponding perception of sleep quality by affecting cognitive and emotional values.
• Lower levels of cognitive-emotional values such as those encountered in the Neutral stage might generate annoyance, yet not sufficient enough to trigger distress reactions. Consequently, annoyance and distress are considered two different concepts in the current framework.
• Cognitive processes for sleep-initiation and sleep-maintenance difficulties are presumed to occur analogously.

Hypothetically, CCF compartments include situation, attention bias, cognitive value (arousal), emotional value (valence), annoyance-distress reaction, and distorted perception. The proposed CCF aims at illustrating how the interaction among cognitive processes contributes to distress reactions.

**FIGURE 1:**
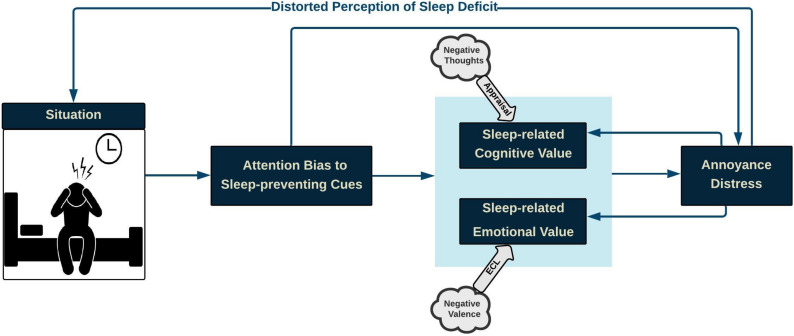
Conceptual Cognitive Framework of Insomnia: During the pre-sleep situation, when attentional resources are captured by insomnia-related stimuli, either directly or through insomnia-related cognitive and emotional values, distress is triggered, thus resulting in a distorted perception of sleep quality, which, in turn, worsens the sleep-initiation process. Likewise, insomnia distress strengthens the negative cognitive-emotional value of difficulty in sleep.

This paper focuses on insomnia experienced before sleep, and the associated “situation” is restricted to the night-time silence period. According to the CCF, when insomnia-related stimuli capture attentional resources, either directly or through corresponding cognitive and emotional values, distress is triggered resulting in a distorted perception. Distress, in turn, feeds back and influences the situation. Similarly, distress reaction fuels back corresponding cognitive and emotional values. The proposed CCF is illustrated in Figure 1.

Toward providing a proof of concept for the proposed CCF, we primarily present some supporting evidence from the Insomnia literature.

## Compartments and Cognitive Processes

### Situation

Nighttime silence in the pre-sleep period can facilitate CAAP of internal (body sensation or thoughts) and external (environmental sounds, light, and heat) stimuli. Bootzin and Rider (1997) noted that “bedtime may often be the first quiet time during the day available to think about the day’s events and to worry and plan for the next day.” Therefore, bed and bedtime tend to be cues for arousal rather than for sleep.

### Attention Bias

Consciously attended internal and external stimuli develop an individual’s predictions and expectations from the pre-sleep situation. Therefore, an attention bias takes place if any novelty or change occurs in the features of such stimuli (Horstmann and Herwig, 2016). Similarly, Roberts et al. (2013) have supported the notion that “discrepancy between an expectation and upcoming stimuli can bias attention” (Horstmann and Herwig, 2016). Additionally, emotion-laden or threat-related stimuli would be prioritized over other stimuli leading to an attentional bias. The same rationale applies to cognitive theories of anxiety disorders (Beck and Clark, 1997), according to which prioritized attention-allocation to threat cues would trigger the development and maintenance of anxiety (Dalgleish and Watts, 1990). Threat cues for patients with insomnia might be related to sleep quality (arising secondarily to bodily sensations such as palpitation, muscle tension, or attention bias toward noises outside and inside the house), which impairs the process of falling asleep.

One of the commonly used paradigms for experimental assessment of attentional bias is the Dot-Probe task. In this, a pair of stimuli (e.g., words or pictures) are presented simultaneously at different locations (up/down or top/bottom) on the screen. The stimuli pair disappear after a fixed time window and a probe appears in the location of emotional (congruent presentations) or neutral (incongruent presentations) stimuli. Subjects are asked to detect and respond to the location of the probe as fast as possible, and the attentional bias is measured through their reaction time in responding to the probe location. A faster probe detection for congruent trials is believed to indicate vigilance, and a slower probe detection for the incongruent trials is suggestive of difficulty in disengaging attention from emotional stimuli (Koster et al., 2004).

Several studies have investigated the impact of the emotional-attentional bias on sleep-related threatening cues through different attentional paradigms, including Dot-probe (MacMahon et al., 2006; Jansson-Fröjmark et al., 2012), flicker (Jones et al., 2005), Posner (Woods et al., 2009), emotional Stroop (Barclay and Ellis, 2013), and eye-tracking (Woods et al., 2013). Most of these studies have endorsed the notion that poor sleepers display attentional bias to sleep-related cues compared with controls. Jansson-Fröjmark et al. (2012) used a dot-probe task to demonstrate that individuals with primary insomnia had a considerably prolonged reaction-time when shifting attention away from insomnia-associated pictures paired with neutral pictures, in comparison to neutral-neutral paired picture presentations as control. Their findings suggest that insomniacs have more difficulty in shifting attention away from insomnia-related stimuli, but are not more vigilant to those stimuli than normal sleepers (Jansson-Fröjmark et al., 2012). However, results reported by Spiegelhalder et al. (2010) yielded no statistically significant preferential attentional-allocation to sleep-related stimuli. Inconsistent results from studies on insomnia may have emerged due to confounding factors and possible bias, impeding their methodologies and study design.

### Emotional Value

The emotional value gets shaped through the evaluative conditional learning (ECL) mechanism which plays a crucial role in liking and disliking stimuli (Ghodratitoostani et al., 2016a, b). Based on ECL, neutral stimuli (CS) can obtain either positive or negative valence after being repeatedly paired with emotion-laden stimuli (US) (De Houwer et al., 2001). Valence represents emotional states varying along a spectrum, ranging from positive to negative feelings with a neutral center-point (Bradley and Lang, 1994). Based on the CCF, CAAP of both CS and US, and their contingencies are required at the time of EC-learning formation. Additionally, evaluative conditioning is an accumulative procedure through which different valenced USs can add to CS valence after being repeatedly paired (Stahl and Unkelbach, 2009). Therefore, EC-learning is resistant to extinction so that neither individual CS/US presence alone, nor pairing CS with different USs would cause the extinction of previously shaped evaluative conditioning (De Houwer et al., 2001). Applying the CCF, the ECL mechanism suggests that the negative valence of other USs fuels a negative sleep-related emotional-value leading to annoyance or distress reaction. Different negative USs can also frequently get paired with internal (bodily sensations) and external (environmental sounds, light, or heat) sleep-preventing stimuli. Thereafter, attending to sleep-preventing cues alone might trigger distress reactions due to the learned USs’ valence.

### Cognitive Value

The cognitive value related to internal and external stimuli is built through an appraisal process. This process initiates when the meaning of an object or event is evaluated in a particular situation according to pre-existing beliefs, desires, and intentions (Scherer et al., 2001). However, not all information but that relevant to individuals’ concerns (Frijda, 1987), can trigger a cognitively aroused state followed by the appraisal. Accordingly, attention bias to sleep-preventing cues (as concern-relevant stimuli) can trigger a cognitively aroused state with subsequent appraisals about insomnia, “*I am never going to get to sleep*,” “*I am not coping with the amount of sleep I get*,” and “*I am going to lose my job*” (Harvey, 2002). Negative thoughts through this appraisal mechanism further fuel the negative sleep-related cognitive value, leading to annoyance or distress reaction.

Self-reported questionnaires are widely used for collecting patients’ thoughts and beliefs about events, situations, or objects that require conscious appraisals of conditions, and their corresponding consequences. Pre-Sleep Arousal Scale (Nicassio et al., 1985), Sleep Disturbance Questionnaire (Espie et al., 1989), and DBAS Scale (Morin, 1993) are commonly applied for assessing thoughts and beliefs related to insomnia. The latter is greatly helpful in clinical practice since it distinguishes salient irrational, and often emotionally loaded thoughts that disturb sleep onset. Nicassio et al. (1985) and Lichstein and Rosenthal (1980) evaluated the intensity of cognitive and somatic arousal at bedtime through the Pre-Sleep Arousal Scale and reported cognitive arousal was more strongly associated with sleeping difficulty. Similarly, Espie et al. (1989) used the Sleep Disturbance Questionnaire and observed “*My mind keeps turning things over*” and “*I am unable to empty my mind*” were the most often endorsed statements among insomniacs (Espie et al., 1989).

Several authors have assessed characteristics of pre-sleep thoughts in terms of content (Harvey, 2000; Wicklow and Espie, 2000), frequency (Barclay and Gregory, 2010), and valence (Kuisk et al., 1989). For instance, Wicklow and Espie (2000) conducted an experimental study on people with clinically significant sleep difficulties using audiotape to record their pre-sleep thoughts and wrist-actigraphy to obtain sleep patterns. The authors indicated that the more frequent thoughts were related to “rehearsing, planning and problem-solving” and “sleep and its consequences,” which strongly correlated with unpleasant emotions and could predict objective sleep latency. Contrarily, Barclay and Gregory (2010) observed that the orientation of catastrophic thoughts in poor sleepers may not be necessarily sleep-specific, instead it was linked to a general tendency to be in an iterative manner regardless of the content or emotional valence. Sleepers were asked to catastrophize their thoughts into three topics namely sleep quality, current personal worries, and hypothetical positive topics. Poor sleepers exhibited greater catastrophic thoughts on every single topic in comparison with good ones, however, no difference was observed in occurrence of catastrophic worry about each topic among poor sleepers. Davey and Levy (1998) suggested that the tendency for repetitive thinking in insomniacs is similar to that of worriers who hold dysfunctional beliefs about the benefits of worrying. In other words, insomniacs believe the ongoing worry helps them find solutions and prevent adverse outcomes. Using DBAS, Morin et al. (1993) reported that not only excessive cognitive activity, but the valence of thoughts also plays a crucial role in provoking emotional reactions to sleep impairment.

### Annoyance-Distress Reaction

Consistent with many cognitive-behavioral studies, the CCF suggests that negative appraisals of insomnia trigger the annoyance-distress reactions. According to the cognitive model of insomnia, excessively negative thinking in the pre-sleep time provokes autonomic arousal, and emotional distress (Harvey, 2002). Tang and Harvey (2004a) have reported that the manipulation of psychological and physiological arousal produces adverse effects on the perception of sleep quality. For illustrative purposes, Baglioni et al. (2010) presented five blocks showing neutral, negative, positive, sleep-related negative and sleep-related positive pictures to evaluate the psychophysiological reactivity to emotional stimuli, both related and unrelated to sleep, in people with primary insomnia and normal sleepers. facial electromyography, heart rate, and cardiac vagal tone were recorded during the picture presentation. The insomnia group indicated an enhanced physiological “craving” response for positive sleep stimuli (e.g., picture of a person asleep in bed), prolonged physiological arousal in response to all stimuli, and increased subjective arousal for negative sleep stimuli (e.g., picture of a person lying awake in bed) when compared to normal sleepers (Baglioni et al., 2010).

### Distorted Perception

According to the CCF, valence and cognitive-arousal as two components of emotion can affect patients’ judgment about sleep quality perception. The following findings lend support to this proposal.

Yoo and Lee (2015) explored the effect of modulating arousal and valence on time-perception in subjects with social anxiety, comparing the time duration of the presented stimuli with the standard duration in training sessions. The perceived duration of negative-stimuli against positive-stimuli was longer with high arousal, but shorter with low arousal levels, suggesting that modifications in the type and magnitude of both valence and arousal modulate time-perception (Yoo and Lee, 2015). This may also be analogous to the distortion in sleep quality-perception in insomniacs.

Using self-reported subjective sleep quality, Tang and Harvey (2004a) observed that experiencing anxious cognitive and physiological arousal in the pre-sleep period resulted in the perception of a longer sleep-onset latency and shorter total sleep time. Moreover, actigraphy results showed contradictions to the reported subjective sleep quality, thus corroborating distorted perception (Tang and Harvey, 2004a).

On the contrary, Herbert et al. (2017) inspected the psychophysiological predictors of subjective/objective sleep discrepancy in Total Sleep Time (Manconi et al., 2010) and Sleep Onset Latency (Herbert et al., 2017) indices among poor sleepers. They reported that excessive pre-sleep cognitive activity and lower mood at the awakening time of the following day are predictors of distortion in time estimation.

### Hypotheses of Conceptual Cognitive Framework

The primary speculation was that the CAAP of internal and external sleep-preventing stimuli captures attentional resources preferentially and triggers the appraisal process, ending with annoyance and distress in the pre-sleep situation. A secondary hypothesis was that intermittent distress experienced in the Clinical stage leads to a misperception about sleep quality.

We applied the multi-mediation insomnia model based on clinical data toward putting the CCF into practice and provide supporting evidence for the proposed causality relationship between the cognitive processes in different stages of insomnia.

## Methods

For the CCF assessment, data were collected from the participants of (1) a randomized crossover three-session double-blind study and (2) an observational prospective cohort study. Both studies were approved by the Ethics Committee for Analysis of Research Projects, Specialized Center of Otorhinolaryngology and Speech Therapy, Hospital das Clínicas de Ribeirão Preto, University of São Paulo, Brazil (HCRP no 55716616.1.1001.5440, and HCRP no 09813519.1.0000.5440; internationally registered with U1111-1236-5441). All participants gave written informed consent.

Two-hundred fifty-three participants (123 female, 130 male) aged 27–72 years (54.43 ± 10.31 years) were recruited for this study. Before the sessions in both studies, participants filled up a Portuguese version of a battery of questionnaires that included (a) a six-item state-trait anxiety inventory (STAI) (Gorenstein and Andrade, 1996) for measuring the presence and severity of anxiety symptoms in the current moment (State anxiety) and a generalized predisposition to be anxious (Trait anxiety), and (b) a mini-sleep questionnaire (MSQ) (Falavigna et al., 2011), i.e., a short screening for sleep disturbances in clinical populations for the assessment of insomnia and sleep difficulties (Table 1). Table 1 shows the items selected from each questionnaire for the development of the insomnia Mediator-Causality model.

**TABLE 1:**
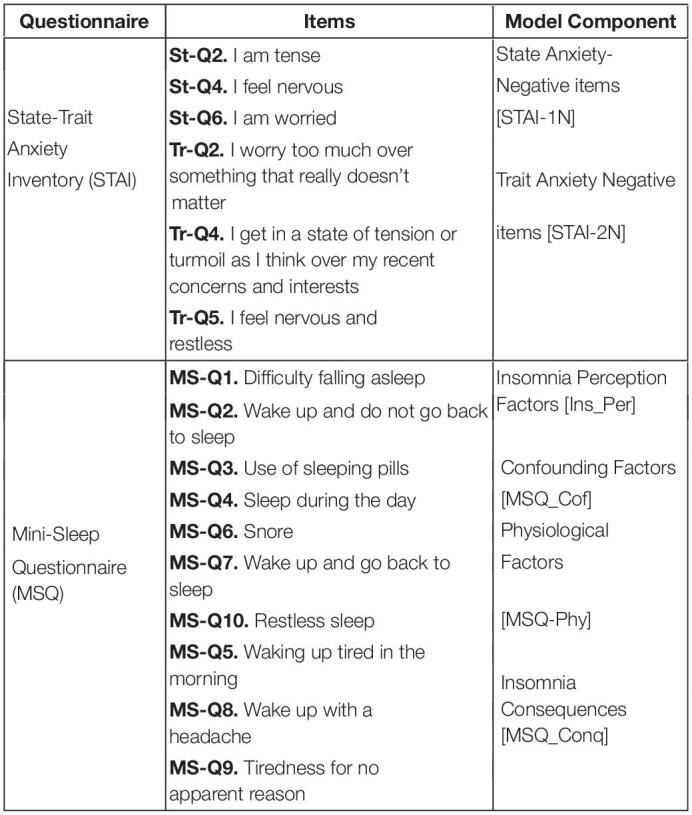
List of selected questions from state-trait anxiety inventory (Gorenstein and Andrade, 1996) and mini-sleep questionnaire (Falavigna et al., 2011) for each model’s component.

### Pre-processing of the Data

**FIGURE 2:**
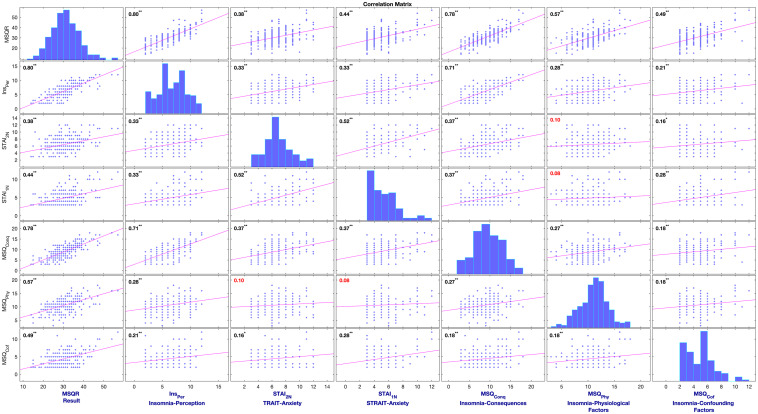
Correlation Matrix of variables used in the multi-mediation model of insomnia to support CCF of insomnia.

The data were anonymized to ensure blinding. Initially, those with missing values were omitted, which resulted in 112 and 134 session-wised questionnaires from the first and second studies, respectively. The datasets were then aggregated and segmented based on the insomnia severity stage. For insomnia, scores of the MSQ-questionnaire lower than 30 (MSQ-R < 30; mild to moderate sleep difficulties) were labeled as Neutral insomnia, and MSQ-R ≥ 30 (severe sleep difficulty) were denoted Clinical insomnia (Natale et al., 2014). Such segmentations provided two sub-datasets (Neutral Insomnia, and Clinical Insomnia) for the statistical analysis. Figure 2 illustrates the correlation matrix of the variables in the mediator model.

### Statistical Analysis

Every segment of the dataset was tested for multicollinearity/autocorrelation by the Durbin-Watson test and showed independence in residuals in general. SPSS v.26 and PROCESS macro (Hayes, 2017) were used for the data analysis. Within the macro, customized models and 5.000 bias-corrected bootstrap samples were set for all tests with the fixed random-seed (“*12020*”). A 95% confidence level was chosen, with significance at for *P* < 0.05 was set. A hierarchical regression analysis investigated the evidence for insomnia CCF within the data-segments, and multiple mediation models were constructed for determining the mediating effects of insomnia-related cognitive items and emotional factors for insomnia. PROCESS macro generated standard errors, *P*-values, and confidence intervals for direct effects, as well as bootstrap confidence intervals for conditional indirect effects.

Datasets and analyzed details are available on “Zenodo” repository with the doi: http://doi.org/10.5281/zenodo.4145224.

### Fundamental Ideas and Postulations for Mediator Models

• Insomnia-perception-factors (Ins_Per) variable contains difficulty in sleep initiation and maintenance.
• The employed dataset was unable to test hypotheses related to the distorted perception of sleep quality.

### Proposed Mediator Model

**FIGURE 3:**
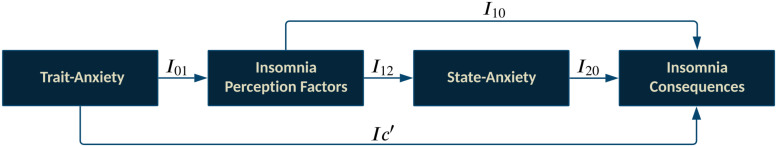
Insomnia mediator model includes the direct effect of trait-anxiety on Insomnia consequences (I_*c’*_); *Ind-1* [*I*_01_ → *I*_10_]: Trait-anxiety → Insomnia perception factors → Insomnia consequences; *Ind-2* [*I*_01_ → *I*_12_ → *I*_20_]: Trait-anxiety → Insomnia perception factors→ State-anxiety→ Insomnia consequences; Covariates: Confounding factors and Physiological problems.

The insomnia mediator model aims to illustrate that negative *trait-anxiety* can affect the perception of deficits in sleep quality. Concurrently, *Insomnia-perception-factors* can directly or through *state-anxiety* affect *insomnia consequences*. The insomnia model is depicted in Figure 3.

Several studies introduced trait-anxiety as an important predisposing factor for both the development and maintenance of insomnia (Sadigh et al., 2014; Bavafa et al., 2018; Lauriola et al., 2019). Harvey (2002) argued that anxious individuals tend to interpret ambiguous situations in a threat-related fashion which, in turn, promotes over-thinking about sleep-related threat cues. This process maintains individuals in a cognitively aroused state which is in contradiction to the relaxed state needed for getting to sleep (Harvey, 2002; Lancee et al., 2017a).

## Results

Multi-mediation regression analysis with the conventional least-squares method revealed that trait-anxiety can only indirectly influence the insomnia consequences. As shown in Figure 3 and Table 2, in the full-dataset, trait-anxiety can lead to insomnia consequences through either insomnia perception, or cascade mediators from insomnia perception to state-anxiety. The 95% confidence interval of bootstrap results revealed “***Ind-1***” [*I*_01_ × *I*_10_ = 0.303] and “***Ind-2***” [*I*_01_ × *I*_12_ × *I*_20_ = 0.025] were significantly different from zero (0.183–0.432) and (0.006–0.057), respectively, but there was not enough evidence for trait-anxiety (*I*_*c*′_ = 0.088, *P* = 0.32) that might directly lead to insomnia consequences.

**TABLE 2:** Mediator model of insomnia in full-dataset.

Paths and Effects	Coefficient	SE	*t*	LLCI	ULCI
Effect of trait-anxiety on insomnia perception factors (*I*_01_ path)	0.363	0.074	4.891	0.217	0.509

Covariate: Effect of confounding factors on insomnia perception factors	0.164	0.075	2.194	0.017	0.311

Covariate: Effect of physiological problems on insomnia perception factors	0.219	0.056	3.903	0.109	0.330

Effect of insomnia perception factors on insomnia consequences (*I*_10_ path)	0.835	0.068	12.308	0.701	0.968

Effect of insomnia perception factors on state-anxiety (*I*_12_ path)	0.239	0.051	4.709	0.139	0.339

Covariate: Effect of confounding factors on state-anxiety	0.241	0.060	4.015	0.123	0.360

Effect of state-anxiety on insomnia consequences (*I*_20_ path)	0.292	0.094	3.120	0.108	0.476

**Direct effect**					

Effect of trait-anxiety on insomnia consequences (*I*_*c*′_ path) [*P*-value = 0.32]	0.088	0.089	0.997	−0.086	0.263

**Bootstrap results for indirect effects**					

Seed number “12020” Bootstrap samples “5000”	**Bootstrap estimate**		**95% confidence interval**		

	**Effect**	**SE**	**Lower**	**Upper**	

Total indirect effect	0.328	0.072	0.196	0.473	

“***Ind-1***”: *I*_01_→*I*_10_	0.303	0.064	0.183	0.432	

“***Ind-2***”: *I*_01_→*I*_12_→*I*_20_	0.025	0.013	0.006	0.057	

According to Table 3, trait-anxiety in the Clinical insomnia segment leads to insomnia consequences only through insomnia perception. The 95% confidence interval of bootstrap results revealed a significant difference in “***Ind-1***” [*I*_01_ × *I*_10_ = 0.161] different from zero (0.040–0.311), but not in “***Ind-2***.” Moreover, there was not enough evidence of trait-anxiety (*I*_*c*′_ = 0.2, *P*-value = 0.104) might directly lead to insomnia consequences.

**TABLE 3:** Mediator model of insomnia in clinical insomnia segment.

Paths and Effects	Coefficient	SE	*t*	LLCI	ULCI
Effect of trait-anxiety on insomnia perception factors (*I*_01_ path)	0.223	0.090	2.480	0.045	0.401

Effect of insomnia perception factors on insomnia consequences (*I*_10_ path)	0.723	0.105	6.863	0.514	0.931

Effect of insomnia perception factors on state-anxiety (*I*_12_ path)	0.227	0.092	2.456	0.044	0.410

Covariate: Effect of confounding factors on state-anxiety	0.254	0.086	2.950	0.084	0.425

Effect of state-anxiety on insomnia consequences (*I*_20_ path)	0.230	0.114	2.013	0.004	0.455

**Direct effect**					

Effect of trait-anxiety on insomnia consequences (*I*_*c*′_ path) [*P*-value = 0.104]	0.200	0.122	1.637	−0.042	0.442

**Bootstrap results for indirect effects**					

Seed number “12020” Bootstrap samples “5000”	**Bootstrap estimate**		**95% confidence interval**		

	**Effect**	**SE**	**Lower**	**Upper**	

Total indirect effect	0.173	0.078	0.042	0.344	

“***Ind-1***”: *I*_01_→*I*_10_	0.161	0.069	0.040	0.311	

“***Ind-2***”: *I*_01_→*I*_12_→*I*_20_	0.012	0.012	−0.000	0.046	

Table 4 shows trait-anxiety neither directly, nor indirectly can lead to insomnia consequences within the Neutral insomnia segment.

**TABLE 4:** Mediator model of insomnia in neutral insomnia segment.

Paths and Effects	Coefficient	SE	*t*	LLCI	ULCI

Effect of trait-anxiety on insomnia perception factors (*I*_01_ path)	0.177	0.109	1.622	−0.042	0.396

Effect of insomnia perception factors on insomnia consequences (*I*_10_ path)	0.881	0.188	4.681	0.503	1.259

Effect of insomnia perception factors on state-anxiety (*I*_12_ path)	0.095	0.113	0.840	−0.131	0.320

Effect of state-anxiety on insomnia consequences (*I*_20_ path)	0.370	0.271	1.365	−0.175	0.914

**Direct effect**					

Effect of trait-anxiety on insomnia consequences (*I*_*c*′_ path) [*P*-value = 0.626]	−0.088	0.178	−0.491	−0.446	0.271

**Bootstrap results for indirect effects**					

Seed number “12020” Bootstrap samples “5000”	**Bootstrap estimate**		**95% confidence interval**		

	**Effect**	**SE**	**Lower**	**Upper**	

Total indirect effect	0.162	0.126	−0.038	0.456	

“***Ind-1***”: *I*_01_→*I*_10_	0.156	0.119	−0.039	0.433	

“***Ind-2***”: *I*_01_→*I*_12_→*I*_20_	0.006	0.015	−0.007	0.049	

### Clinical Implications

Insomnia *per se* is a clinical issue with short-term and long-term consequences affecting both physiological and psychological systems. It has been associated (at least partly in terms of duration and severity) with increasing the incidence and worsening the state of many pre-existing clinical conditions (Schutte-Rodin et al., 2008). Hence, the need for formulating a conceptual and concurrently pragmatic framework toward an individualized approach for people suffering from insomnia.

The proposed CCF, together with insomnia mediator models, explained the contribution of cognitive processes to the development and maintenance of clinical insomnia. The CCF proposes the following predictions and target-oriented clinical implications:

#### Decreasing Attentional Bias

Conceptual cognitive framework predicts that attentional bias modification (ABM) training can decrease the attentional bias to insomnia. In practice, the ABM treatment encourages the insomniacs to shift attention away from the negative sleep-related words toward a neutral one, thus reducing attention bias toward sleep-related threatening cue. Such a simple task enables patients to consciously and repeatedly select unbiased information over negative information, thereby progressively help to develop a tendency to not focus on negative information related to insomnia in their daily life (Clarke et al., 2016).

Milkins et al. (2016) conducted a crossover study in which 18 insomniacs alternatively fulfilled an ABM task and a non-ABM control task before sleep across six successive nights. At nights on which the subjects performed the ABM task, they reported shorter sleep-onset latencies and lower pre-sleep worry, than the nights on which they performed the control task. Likewise, in a parallel design (Clarke et al., 2016), 36 students with sleep problems underwent ABM or control training sessions across five nights. Compared with the control condition, subjects who underwent ABM training reported less pre-sleep arousal, fell asleep faster, woke less often during the night, reported better overall sleep quality, and had significant reductions in sleep-related anxiety (Clarke et al., 2016). These findings support the above-mentioned proposition.

In contrast, the results of Lancee et al. (2017b) showed no added benefit of the ABM training over the placebo training on sleep-related indices and outcome measures. The authors believe it was probably due to the absence of attentional bias at baseline and hence no change could be deduced after training (Lancee et al., 2017b).

#### Employing Attention-Distraction Techniques Can Help Deviate Attention From Concerns-Relevant Topics to Neutral Ones

Addressing the issue of attentional bias toward relevant topics, Haynes et al. (1981) observed that engagement with a challenging mental arithmetic problem reduced subjective sleep latency among insomniacs (Haynes et al., 1981). Similarly, practicing crossword puzzles, reading, and listening to audiobooks could provide sufficient distraction so that the patient would no longer attend to or think about their inability to sleep. Troxel et al. (2012) recommended patients should keep doing those activities until they feel sleepy enough to return to bed. And, if they cannot fall asleep after returning to bed, the process should be repeated (Troxel et al., 2012).

#### Preventing Annoyance and Distress-Reaction

Conceptual cognitive framework draws attention to the crucial role of appraisal and ECL mechanisms in reducing negative cognitive and emotional value.

##### Cognitive-behavioral therapy (CBT) to reduce the negative cognitive-value related to insomnia

Sleep difficulties are commonly accompanied by dysfunctional beliefs, unrealistic expectations, and worries, which contribute to distress and maladaptive sleep habits producing an anxious state opposite to the relaxation required for sleeping. Therefore, patients’ beliefs regarding sleep and insomnia must be explored and attempts be made to change them eventually. Cognitive therapy aims at the identification of dysfunctional beliefs and attitudes related to sleep and their replacement with more adaptive substitutes. Cognitive therapies also address catastrophizing the consequences of poor sleep to help patients reconceptualize the realities of their beliefs, thereby reducing the upcoming distress and arousal that impedes sleeping (Perlis et al., 2006). Through cognitive-behavioral therapy (CBT) a combination of cognitive reconstruction and behavioral techniques are delivered to encourage patients to develop more adaptive coping skills and stop self-criticizing (Perlis et al., 2006). The European guideline for diagnosis and treatment of insomnia (Riemann et al., 2017) recommends CBT for insomnia (CBT-I) as the first-line of treatment for chronic insomnia.

Furthermore, several systematic reviews and meta-analyses (Taylor and Pruiksma, 2014; Mitchell et al., 2019) have reported strong empirical support for CBT-I on different subjective and objective sleep parameters. CBT-I’s common approaches for non-comorbid insomnia were cognitive therapy, stimulus control, sleep restriction, sleep hygiene, and relaxation. The results indicated that CBT-I improved sleep onset latency, wake after sleep onset, total sleep time, and sleep efficiency. The changes persisted over time alleviating the symptoms (Wang et al., 2005; Trauer et al., 2015).

##### Mindfulness-based cognitive therapy (MBCT) for reducing the negative cognitive and emotional value related to insomnia

Mindfulness-based cognitive therapy (MBCT), as an emotion-regulation based psychotherapy, is a purposeful and unbiased form of therapy directing attention to the present moment as a way of self-regulation that promotes mind-body relaxation (Ludwig and Kabat-Zinn, 2008). The approach educates people toward changing their relationship with their thoughts and negative emotions. Patients must be aware of their thoughts and are inspired to take a non-judgmental perspective on them rather than a negative, self-referential assessment that intensifies both negative thoughts and emotions (Ludwig and Kabat-Zinn, 2008). In concordance with suggestions put forth by the CCF, Shallcross and Visvanathan (2016) have explained that experiential awareness, attentional control, and acceptance techniques used in MBCT interventions improve rumination, arousal, selective attention, and the distorted perception involved in the development and maintenance of insomnia (Shallcross and Visvanathan, 2016).

The MBCT protocol tailored for insomniacs showed significant pre–post improvements in self-reported total sleep time and various thought-control domains, along with reductions in sleep-related monitoring and worry (Heidenreich et al., 2006). MBCT was also effective for individuals with a history of depression or anxiety accompanied by sleep difficulty or insomnia (Ree and Craigie, 2007; Yook et al., 2008; Britton et al., 2010). Ree and Craigie (2007) reported decreased scores of insomnia severity symptoms lasting for about 3 months with the MBCT. Similarly, MBCT protocol in older adults showed a 14.5% improvement in self-reported sleep problems (Foulk et al., 2014). Self-regulation of attention and orientation to experience to achieve better sleep are the proposed mechanisms of actions for MBCT (Larouche et al., 2014). A recent comprehensive meta-analysis reported significantly improved insomnia symptoms as measured by the Pittsburgh Sleep Quality Index (Wang et al., 2020).

##### ECL mechanism for modifications in negative emotional-value related to insomnia

Positive emotion-induction techniques can reduce the negative valence of insomnia when paired with positively valenced and high arousal pictures, films (Uhrig et al., 2016), audio (Bergman et al., 2016), music, and video clips (Lazar and Pearlman-Avnion, 2014; Siedlecka and Denson, 2019). Game-like design, app-based format, goggles of virtual reality, or a screen are different ways to present stimuli to provide cost-effective home-based individualized treatments.

#### Rectifying the Distorted Perception of the Quality of Sleep Deficit

Digital-technology approaches are believed to provide an online measurement of sleep duration and correct the distorted perception of sleep deficit. Since we have established that negative emotions might influence the perception of insomniacs about their sleep deficit, interventions aiming at emotion-regulation or modifications of dysfunctional beliefs may help prevent the formation of the distorted perception. Furthermore, Holter monitoring of rest/activity cycle of sleep, smartphone gadgets (Izmailova et al., 2018), actigraphy, and sleep diary (Tang and Harvey, 2004b) might help insomniacs correct misperception. In contrast, parts of the literature studying the time-perception concept (Thomas and Cantor, 1975, 1976) have revealed that when more information is processed, time is perceived as longer. A high level of cognitive arousal and repetitive thought patterns distorts time perception for insomniacs, leading to an overestimation of sleep onset latency (Tang and Harvey, 2004a). Another implication is that during sleep onset, cognitive arousal maintains an enhanced sensory and memory processing level obscuring the distinction between sleep and wakefulness (Perlis et al., 1997).

### Future Trends

The clinical recommendations provided in this paper can be applied separately or in combination, to plan treatment for individuals with insomnia. The CCF builds upon a general assumption that patients should be consciously and actively involved in the rehabilitation process. Subsequently, new treatments can be developed aimed at encouraging patients to be consciously aware of their negative thoughts related to sleep-difficulty and contingencies for intervention. Moreover, the inclusion of surrogate measurements is recommended for guaranteeing the patient’s conscious attended-awareness. Collectively, the CCF can provide a decision-support platform for clinicians to deliver more targeted interventions, and eventually, the methodologies suggested can provide a reliable platform to build a CCF for other cognitive disorders and support the causal clinical data models. This novel approach can improve our knowledge of psychological disturbances and complex comorbidities toward the design of rehabilitation interventions and suggestions in line with the “preventive medicine” policies.

### Limitation

The CCF of insomnia, its predictions, and the corresponding suggested interventions do not include patients with organic sleep disorders, general cognitive distortion, and psychotic problems. MSQ was obtained from patients with complaints of tinnitus at the clinic during the day, and not before sleep. Despite their importance, daytime cognitive processes were not taken into account in the presented framework. Lastly, to achieve clinical endpoints, repeated measures and longitudinal studies are required to improve predictabilities.

## Data Availability Statement

The datasets used and analyzed during this study are available from the corresponding author on reasonable request and filling out NEL-Consent redirecting to “Zenodo” with the doi: http://doi.org/10.5281/zenodo.4145224.

## Ethics Statement

The studies involving human participants were reviewed and approved by Ethics Committee for Analysis of Research Projects, Specialized Center of Otorhinolaryngology and Speech Therapy, Hospital das Clínicas de Ribeirão Preto, University of São Paulo, Brazil (HCRP no. 55716616.1.1001.5440, and HCRP no. 09813519.1.0000.5440; internationally registered with U1111-1236-5441). All subjects gave written informed consent prior to participation in the study.

## Author Contributions

ZV: leading author responsible for manuscript development, concept and study design, conceptual modeling participation, and data acquisition in the clinic. MN: collaborating in manuscript development and concept. JL: supervising clinical data acquisition and conceptual development, and collaborating in manuscript development. AD: collaborating in manuscript development and supervising data mining and modeling. MH: monitoring clinical data acquisition, and collaborating in manuscript development. IG: manuscript development, concept and study design, analogy, numerical methodology design and implementation, and data acquisition, and data pre-processing. All authors read and approved the final manuscript.

## Conflict of Interest

The authors declare that the research was conducted in the absence of any commercial or financial relationships that could be construed as a potential conflict of interest.

## Abbreviations

ABM: attentional bias modification
BIISS-RESO: behaviorally induced insufficient sleep syndrome with restricted and extended sleep opportunity
CAAP: conscious attended awareness perception
CBT: cognitive-behavioral therapy
CBT-I: cognitive-behavioral therapy for insomnia
CCF: conceptual cognitive framework
CEOF: Centro Especializado de Otorrinolaringologia e Fonoaudiologia
CS: conditioned stimulus
CVMIMD: cognitive vulnerability model for insomnia induced mood disturbances
DBAS: dysfunctional beliefs and attitudes about sleep
ECL: evaluative conditional learning
EDS: excessive daytime somnolence
GCVI: global cognitive vulnerability to insomnia
Ind: Indirect
MBCT: mindfulness-based cognitive therapy
MSQ: mini-sleep questionnaire
MSQ-R: mini-sleep questionnaire result
PFC: prefrontal cortex
SSCV: sleep-specific cognitive vulnerability
STAI: state-trait anxiety inventory
US: unconditioned Stimulus
WASO: wakes after sleep onset.
